# World’s Columbian Dental Congress

**Published:** 1893-03

**Authors:** 


### WORLD'S COLUMBIAN DENTAL CONGRESS.—NEW ORDER OF BUSINESS.

We publish on another page the order of business as arranged for the Dental Congress. Had it not been received from the constituted authority we should have been tempted to regard it in the light of a burlesque. Whoever arranged it must have supposed that the making of addresses constitutes the chief end of man, for we find every day, excepting Thursday, at exactly 12 m., an "Address before the whole Congress" is in order. On that day (Thursday), in addition to the 12 m. address, the members are to be edified in the evening by a "public address, under direction of World's Congress Auxiliary." It is not surprising, after this wordy deluge, that on Saturday the members are expected to lunch "in the restaurant."

On Wednesday, at 8 P.M., there is to be a lantern exhibit, and at the same hour, 8 P.M., "Conversazione." Are we to understand that the members are to *converse during the exhibit?*

There is no disposition to assume a spirit of criticism, but it does seem that a better arrangement could be made. The time given to addresses should be limited to the first day and the first session, and the other periods to scientific matters. The time is all too short, and the programme, as at present arranged, allows none for discussion, as it will, doubtless, require all of the hours for the reading of the papers presented.

